# The complete mitogenome of the blackgill catshark, *Parmaturus melanobranchus* (Chan, 1966)

**DOI:** 10.1080/23802359.2021.1951136

**Published:** 2021-07-15

**Authors:** Wenjun Chen, Xiaogu Wang, Xiangxing Gao, Xiaohui Gao, Chen Fang, Chenhong Li

**Affiliations:** a Key Laboratory of Marine Ecosystem Dynamics, Second Institute of Oceanography, Ministry of Natural Resources, Hangzhou, China; b Shanghai Universities Key Laboratory of Marine Animal Taxonomy and Evolution, Shanghai Ocean University, Shanghai, China; c Shanghai Collaborative Innovation for Aquatic Animal Genetics and Breeding, Shanghai Ocean University, Shanghai, China; d National Deep Sea Center, Qingdao, China; e Hangzhou Haikuoyu Ecological Technology Co., Ltd, Hangzhou, China

**Keywords:** *Parmaturus melanobranchus*, catshark, mitogenome, phylogenetic analysis

## Abstract

A rare specimen of *Parmaturus melanobranchus* was collected from the South China Sea. The complete mitochondrial genome of the specimen was sequenced using Illumina Hiseq platform and assembled with Geneious and Trinity. The mitogenome is 16,687 bp long with a base composition of 30.4% A, 14.1% G, 23.5% C and 32.1% T, respectively. A total of 37 genes were predicted containing 13 protein-coding genes, 2 ribosomal RNA genes and 22 transfer RNA genes. This is the first complete mitochondrial genome published of this species.

A specimen of a catshark was collected at 20°59′N, 117°56′E between the coast of Taiwan and Hongkong, South China Sea at a depth of 1302 m and an ambient temperature of 3.2 °C. The specimen was deposited with a voucher number of B3416400001 at Second Institute of Oceanography (Xiaogu Wang, wangxiaogu1968@126.com), Hangzhou, China. And a tissue sample was deposited at the Lab of Molecular Systematics and Ecology, Shanghai Ocean University, Shanghai, China. The specimen was identified as *Parmaturus melanobranchus*, a rare species of cat shark, based on the identification criteria defined by Chan et al. (1966 ) and Lee and Shao (2010). There have been only four specimens collected since its first description in 1966 (Lee and Shao 2010). Meanwhile there is only one short mitochondrial sequence of 12S rRNA (181 bp) available on GenBank for this species (GenBank accession number: LC020831, accessed on 17 June 2021).

Total DNA was extracted using the Ezup Column Animal Genomic DNA Purification Kit (Catalog number: B518251-0100) from Sangon Biotech (Shanghai, China). An Illumina library was prepared following Meyer and Kircher (2010) and the mitochondrial DNA was enriched and sequenced using an Illumina Hiseq platform. Geneious 7.1.9 (https://www.geneious.com) and Trinity (Grabherr et al. 2011) were used for read assembly. MITOS (Bernt et al. 2013) was used for the annotation after obtaining complete sequence of the mitochondrial genome.

The mitochondrial genome of *P. melanobranchus* was 16,687 bp in length and comprised 13 protein-coding genes, 2 ribosomal RNA genes and 22 transfer RNA genes. Twenty-eight of the 37 genes were found on the heavy (H) strand and 9 genes were on the light (L) strand. The base composition of the mitogenome was 30.4% A, 14.1% G, 23.5% C and 32.1% T, with a G + C content of 55.6%.

There was no previous complete mitochondrial genome of this species or genus available on GenBank. We downloaded all the available mitochondrial data of this genus and reconstructed a supermatrix tree using MEGA X (see details and GenBank numbers in Figure 1(A)) (Kumar et al. 2018). The results showed that the mitogenome was grouped with the 12S rRNA sequence of *P. melanobranchus* (LC020831) collected by the Okinawa Churaumi Foundation. Actually there is only one-nucleotide difference between the 181 bp sequence of 12S gene of these two specimens. We also reconstructed a phylogenetic tree of the 13 protein-coding genes (PCGs) of 9 sharks (see details and GenBank numbers in Figure 1(B)) using MEGA X. The results showed that our mitogenome was grouped with the catshark family. This is the first complete mitochondrial genome published of this species and genus, and will likely help phylogenetic analysis of sharks using complete mitochondrial genomes. In addition, the recorded depth of the specimen extends the vertical distribution from 1100 m (Rigby et al. 2020) to 1300 m.

**Figure 1.**
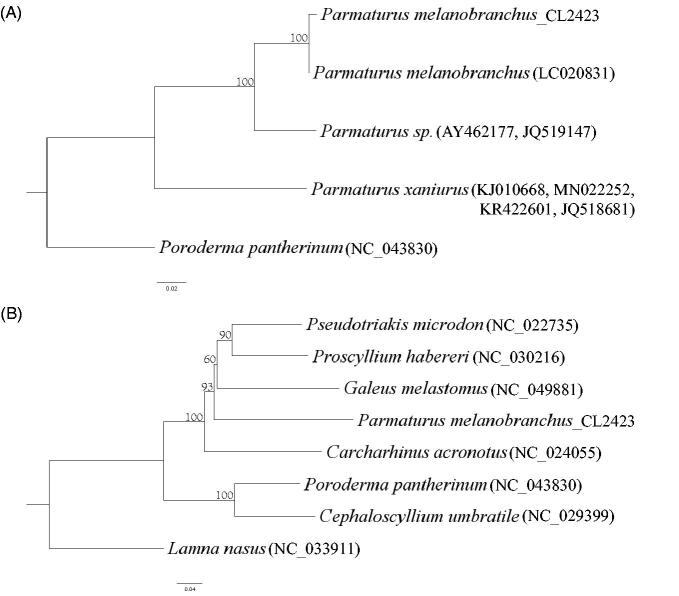
Phylogenetic analysis. (A) Phylogenetic supermatrix tree reconstructed using MEGA X. (B) Phylogenetic tree reconstructed with 13 protein-coding genes of 8 sharks using MEGA X.

## Data Availability

The data that support the findings of this study will be available in GenBank at https://www.ncbi.nlm.nih.gov/, accession number MZ018622. The associated BioProject, SRA, and Bio-Sample numbers are PRJNA735909, SRS9155331, and SAMN19602241, respectively.

## References

[CIT0001] Bernt M, Donath A, Jühling F, Externbrink F, Florentz C, Fritzsch G, Pütz J, Middendorf M, Stadler PF. 2013. MITOS: improved de novo metazoan mitochondrial genome annotation. Mol Phylogenet Evol. 69(2):313–319. 22982435 10.1016/j.ympev.2012.08.023

[CIT0002] Chan WL. 1966. New sharks from the South China Sea. J Zool. 148(2):218–237.

[CIT0003] Grabherr MG, Haas BJ, Yassour M, Levin JZ, Thompson DA, Amit I, Adiconis X, Fan L, Raychowdhury R, Zeng Q. 2011. Trinity: reconstructing a full-length transcriptome without a genome from RNA-Seq data. Nat Biotechnol. 29(7):644–652. 21572440 10.1038/nbt.1883 PMC3571712

[CIT0004] Kumar S, Stecher G, Li M, Knyaz C, Tamura K. 2018. MEGA X: molecular evolutionary genetics analysis across computing platforms. Mol Biol Evol. 35(6):1547–1549. 29722887 10.1093/molbev/msy096 PMC5967553

[CIT0005] Lee PF, Shao KT. 2010. New record of the rare shark *Parmaturus melanobranchius* (Scyliorhinidae) from Taiwan. Taiwania. 55(4):386–390.

[CIT0006] Meyer M, Kircher M. 2010. Illumina sequencing library preparation for highly multiplexed target capture and sequencing. Cold Spring Harb Protoc. 2010(6):pdb.prot5448. 20516186 10.1101/pdb.prot5448

[CIT0007] Rigby CL, Chen X, Ebert DA, Herman K, Ho H, Hsu H, Zhang J. 2020. *Parmaturus melanobranchus*. The IUCN Red List of Threatened Species 2020: e.T161497A124495582. 10.2305/IUCN.UK.2020-3.RLTS.T161497A124495582.en. Downloaded on 03 June 2021.

